# Research on heavy metal level and co-occurrence network in typical ecological fragile area

**DOI:** 10.1007/s40201-021-00625-w

**Published:** 2021-03-08

**Authors:** Yiwei Zhao, Liangmin Gao, Fugeng Zha, Xiaoqing Chen, Xiaofang Zhou, Xinfu Wang, Yang Chen, Xiangwei Pan

**Affiliations:** Department of Environmental Science and Engineering, School of Earth and Environment, Anhui University of Science and Technology, 232001 Huainan, People’s Republic of China

**Keywords:** Heavy metals, Co‐occurrence network, Geostatistical analysis, Moran Index, Soil

## Abstract

Due to the special sensitivity of typical ecologically fragile areas, a series of human life, mining, and other activities have a greater impact on the environment. In this study, three coal mines in Ordos City on the Loess Plateau were selected as the study area, and the pollution levels of heavy metals in the area were studied by measuring As, Hg, Cr, Cd, Cu, Ni, and Pb in the soil of 131 sampling points. Combined with the concept of “co-occurrence network” in biology, the level of heavy metals in soil was studied using geostatistics and remote sensing databases. The results showed that the concentrations of Hg, Cr, Ni, Cu, and Pb in more than half of the sampling points were higher than the local environmental background value, but did not exceed the risk control value specified by China, indicating that human factors have a greater influence, while Cd and As elements are mainly affected Soil parent material and human factors influence. Heavy metal elements have nothing to do with clay and silt but have an obvious correlation with gravel. Cd, Pb, As and Ni, Cd, Cr are all positively correlated, and different heavy metals are in space The distribution also reflects the autocorrelation, mainly concentrated in the northeast of the TS mining area and the middle of the PS mining area.

## Introduction

 Soil heavy metal pollution is one of the main environmental problems related to potential ecological risks and public health impacts [1]. Due to the toxicity and refractory of heavy metals, over time, the accumulation of heavy metals in the soil will lead to soil nutrients. Loss, leading to degradation of soil biology and function [2]. Heavy metals enter the human body through a variety of ways (for example, ingestion of soil, inhalation of dust, skin contact with soil, and consumption of food crops grown in contaminated soil), increasing the risk of cardiovascular, neurological, and kidney diseases [3–6].

Relevant studies have shown that the accumulation of heavy metals is related to location and has obvious spatial inhomogeneity [7]. In areas of severe human disturbance, especially mining and smelting areas, the concentration of heavy metals is relatively high [2, 8–10]. The concentration of most heavy metals in the soil of Inner Mongolia is higher than the average background value. In particular, the high coefficient of variation of Ge (1.03) and As (0.56) indicate that the presence of open-pit mines affects the concentration of these elements. At the junction of rivers and valleys and near mining areas, the comprehensive ecological risk of heavy metals reaches level III, while the comprehensive ecological risk levels of Zhaosu River are A and B. It can be considered that mining activities and mineral adsorption are the main environmental impacts of arsenic and mercury. Control factors [11–14]. Research on the agricultural soil around a manganese mine in Guangxi shows that the soil is polluted by elements such as manganese, lead, zinc, and cadmium (Cd) [15]. However, the research on heavy metals in soil is mostly carried out on a mine scale, and there are relatively few studies on the influence of soil heavy metals in plateau areas [16].

The ES mine, TS mine area, and PS mine area are located in the Inner Mongolia section of the Yellow River Basin. The Inner Mongolia section of the Yellow River lies between the southern foot of the Yinshan Mountains and the Ordos Plateau, passing through the Ulan Buh Desert, Hetao Irrigation District, and Tumochuan Plain and Kubuqi Desert., The overall ecological environment of the basin area is fragile, and it is a concentrated area of ​​ecological environment management. The ecological environment problems in some areas are prominent. The area of ​​desertified land in 7 league cities in the basin accounts for about 60 % of the area of ​​desertified land in the whole district, and the area of ​​desertified land is about 72 % of the area of ​​desertified land in the whole district, which has caused serious adverse effects on lakes, transportation routes, farmland, and urban development. At the same time, most of the energy, chemical, and metallurgical industries in the region are distributed in the basin, with prominent structural and structural pollution hazards [17, 18], and great pressure on environmental pollution control in the basin [19]. The main goals of this research are: (i) to determine the degree of pollution of heavy metals in the soil; (ii) to visualize the internal relationship between heavy metals and soil texture based on the concept of “co-occurrence network” in biology; (iii) based on geostatistics and PMF The model determines the spatial variation characteristics of soil heavy metal concentrations and explains the source of pollution. As far as we know, this idea has not been used to evaluate and explain heavy metal pollution in soils in typical ecologically fragile areas.

## Materials and methods

### Study area

**Fig. 1:**
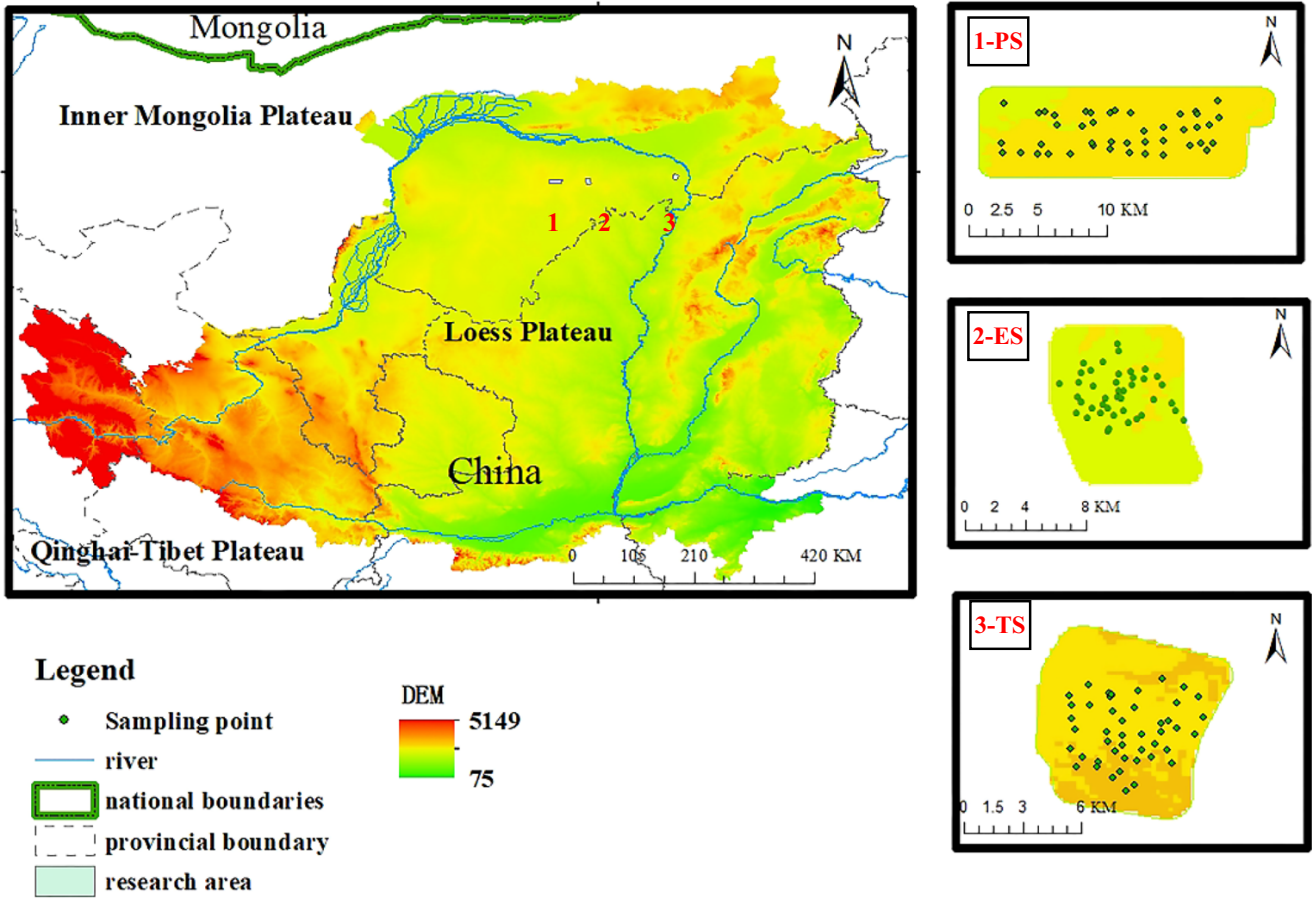
Location map of the study area

To evaluate the level of heavy metals in typical ecologically fragile areas, the study selected the northern part of the Loess Plateau (ES mine, PS mine, TS mine) as the study area (see Fig. 1). The study area is located between 39°~40°N latitude and 112°~119°E longitude. It is located at the junction of the Ordos Plateau, the Loess Plateau, and the Kubuqi Desert. It is a transitional zone of blast circulation and poor hydrothermal conditions. It is a typical ecologically fragile area. The administrative division belongs to Ordos City, Inner Mongolia. Among them, the ES No. 2 minefields are located in Hantai Town, Dongsheng District, Ordos City, Inner Mongolia Autonomous Region. The PS research area is under the jurisdiction of Borjianghaizi Town, Dongsheng District, Ordos City. The TS research area is located in the Inner Mongolia Autonomous Region. The territory of Xuejiawan, Zhungeer Banner. The soil types in the project area mainly include sandy loess and coarse bone soil. The soil parent materials mainly include alluvial deposits, residual slope deposits, and a small amount of secondary loess and aeolian sand. Natural resources are rich, especially coal resources have proven reserves of more than 200 billion tons, accounting for about 1/6 of the country. GuangxiXing studied soil profiles from China’s Inner Mongolia region and found that heavy metals such as Ag are significantly enriched in the surface soil caused by industrial activities [20]. Li Yuanjie systematically analyzed the soil heavy metals in the western Inner Mongolia Autonomous Region with the pollution index method. The results show that the horizontal spatial distribution of pollutant components is affected by short-distance migration and transportation, and the vertical distribution is controlled by runoff [21].

### Data and methods

**Fig. 2:**
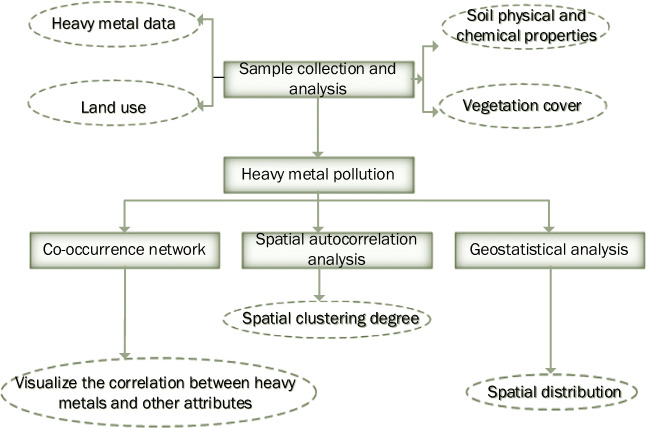
Flow chart of research content

To systematically evaluate the soil heavy metal levels in typical ecologically fragile areas, the study collected soils from 131 sampling points in ES mine, PS mine, and TS mine. GPS was used to locate the sampling location. The samples are thoroughly mixed to form a composite sample. All soil samples are stored in plastic bags on ice, placed in a portable cryostat, and then immediately transported to the laboratory, freeze-dried, ground, and passed through a 100-mesh sieve. After digestion, the concentration of As, Hg, Cr, Ni, Cu, Cd, and Pb is determined by ICP-MS. When the sampling amount is 0.1 g and the fixed volume after digestion is 50 ml, Cd, Cu, Cr, Ni, Pb, The detection limits of As method are 0.07 mg/kg, 0.5 mg/kg, 2 mg/kg, 2 mg/kg, 2 mg/kg, 0.6 mg/kg, and the lower detection limits are 0.28 mg/kg, 2.0 mg/kg, 8 mg/kg, 8 mg/kg, 8 mg/kg, 2.4 mg/kg. When the sampling volume is 0.5 g, the detection limit of the Hg method is 0.002 mg/kg, and the lower limit of determination is 0.008 mg/kg. Quality assurance and control are carried out by evaluating the metal concentration in blank samples and repeated samples based on the standard reference materials obtained from the China National Standard Reference Material Center. A BT-2003 laser particle size distribution analyzer was used to determine the particle size distribution (Fig. 2).

### Descriptive statistics

The application software origin2018 clarifies the concentration range of heavy metal levels in the study area and is used to preliminarily judge the pollution of each heavy metal indicator.

### Co‐occurrence networks

Visualize the correlation between heavy metals and other soil properties by using the social analysis network generated by Gephi 0.9.2. Using different statistical algorithms and combining the connection between nodes and edges, the overall characteristics and modularity of the network, the centrality, dynamicity, and path characteristics of the nodes are calculated in different ways. The data obtained by the pre-processing is stored in the graphic data and then displayed on the graphic appearance. In terms of generating maps, the specific purpose of the co-occurrence network is to generate an interactive interface to help visualize statistical information and further search for the same source of pollution of heavy metals in the soil from the level of concentration values. In the network involved in this article, they are all undirected graphs (the direction is not mentioned). Among them, the physicochemical properties of the soil and the state of heavy metals are indicated by the nodes in the graph, and the edges connecting the nodes represent the internal relationship between the nodes, and their thickness (Pearson correlation coefficient,PCCs ) are positively correlated with the strength of the relationship, with a significance level of 0.05.

### Spatial autocorrelation and geostatistical analysis

In this study, Open Geoda was used for the statistical analysis of the spatial autocorrelation of heavy metals. Moran’s I coefficient was used to reflect the degree of clustering at the spatial level. Unlike the Pearson correlation coefficient, the spatial autocorrelation coefficient is correlated with the observation criterion (or the size of the analysis). Before spatial automatic correlation analysis, we have finished the line normalization of the weights to ensure that the index value was normalized to the range of -1.0 ~ 1.0 by the variance. Moran’s I statistics show:

$$I=\frac{n}{{S}_{0}}\frac{\sum _{i=1}^{n}{\sum }_{j=1}^{n}{\omega }_{i,j}{Z}_{i}{Z}_{j}}{\sum _{i=1}^{n}{z}_{i}^{2}}$$

Where *Z*_*i*_ is the deviation of the characteristic of element *x*_*i*_ from its average value $\stackrel{-}{x}$, *Z*_*j*_ is considered to be the deviation of element *x*_*i*_ and its average value $\stackrel{-}{x}$, ${\omega }_{i,j}$ is the spatial weight between the elements i and j, n is the total number of elements, and S_0_ is the summation of all spatial weights:

$${S}_{0}=\sum _{i=1}^{n}\sum _{j=1}^{n}{\omega }_{i,j}$$

The statistical *z*_*i*_ score is:

$${z}_{i}=\frac{i+\frac{1}{n-1}}{\sqrt{E\left[{i}^{2}\right]-{E\left[i\right]}^{2}}}$$

Among them, *E*[*i*] represents the expected value of Moran’s I. When the value of Moran’s I is greater than 0, it shows that there is a positive spatial correlation in the study area, whereas when it is less than 0, there is a negative correlation, and the absolute value close to 1 indicates that the spatial correlation is stronger. In this study, they showed that there may be a source of pollution in the high-value area. Combined with the statistical analysis of the area, the concentration value exceeded the standard, and the high-value area of Moran’s I was the source of pollution.

We performed the concentration difference using the empirical Bayesian Kriging method in ArcGIS 10.2.

## Results and discussion

### Heavy metal concentrations

**Fig. 3:**
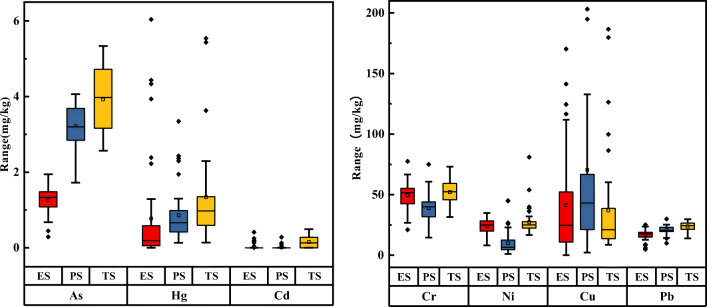
Heavy metal levels in soil in three mining areas

The content of arsenic in the three’s soil mining areas from Fig. 3 gradually increased (c(ES) > c(PS) > c(TS)). Among them, the distribution of arsenic in the ES mining area was left-skewed, and the data was concentrated at 1 mg/kg ~ 1.5 mg/kg, the arsenic content of the PS and TS mining areas is normally distributed, and the overall content is between 2 mg/kg ~ 6 mg/kg, and the environmental background value specified that by the state is above it. The distribution of mercury in the three mining areas is correct. Of these, 14 % and 9 % of foreigners are in the ES and PS mining areas, respectively. The overall content is between 0 ~ 2 mg/kg, 91 % of the data exceeds the local environmental background value, and the maximum value is close to 6 mg/kg. The distribution of cadmium in the ES and PS mining areas is positively skewed, concentrated between 0 ~ 0.5 mg/kg, and the data is relatively concentrated, both of which are lower than the local environmental background value(Table 1). The distribution in the TS mining area is normally distributed, the median and the average are at the same level. The distribution of chromium in the three mining areas is normal, 70 % of the data exceeds the local environmental background value, and the maximum value is close to 75 mg/kg.

**Table 1:** Standard values of heavy metals in soil in Inner Mongolia

	As	Hg	Cr	Ni	Cu	Cd	Pb
Environmental background value^a^	7.5	0.04	41.4	19.5	14.1	0.053	17.2
Second level standard value	70	20	1000	200	500	20	600

^a^ Environmental background value, the data comes from “Chinese soil element background value”, the unit is mg/kg

Nickel in the mining’s soil area is in a right-skewed distribution, and the whole is between 0 ~ 35 mg/kg, of which 60 % of the data is higher than the first-level limit specified by the state, but does not exceed the second-level limit, indicating that there is no pollution hazard, Can be called “still clean”. The copper data are scattered, The maximum minus the minimum reaches nearly two hundred, the mean value is significantly higher than the average, and the curve is strong. Only 25 % of the data meets the local environmental background value, but it is still within the secondary range (500 mg/kg). The lead data is relatively concentrated, with a positively skewed distribution, and the skewness is weak. The data float around 25 mg/kg. There are 80 % of the data that exceed the local environmental background value and are relatively less affected by the natural background value. Based on the analysis of the above data, the level of heavy metals in the study area is high, and human factors have a greater influence.

### Co‐occurrence network analysis

**Fig. 4:**
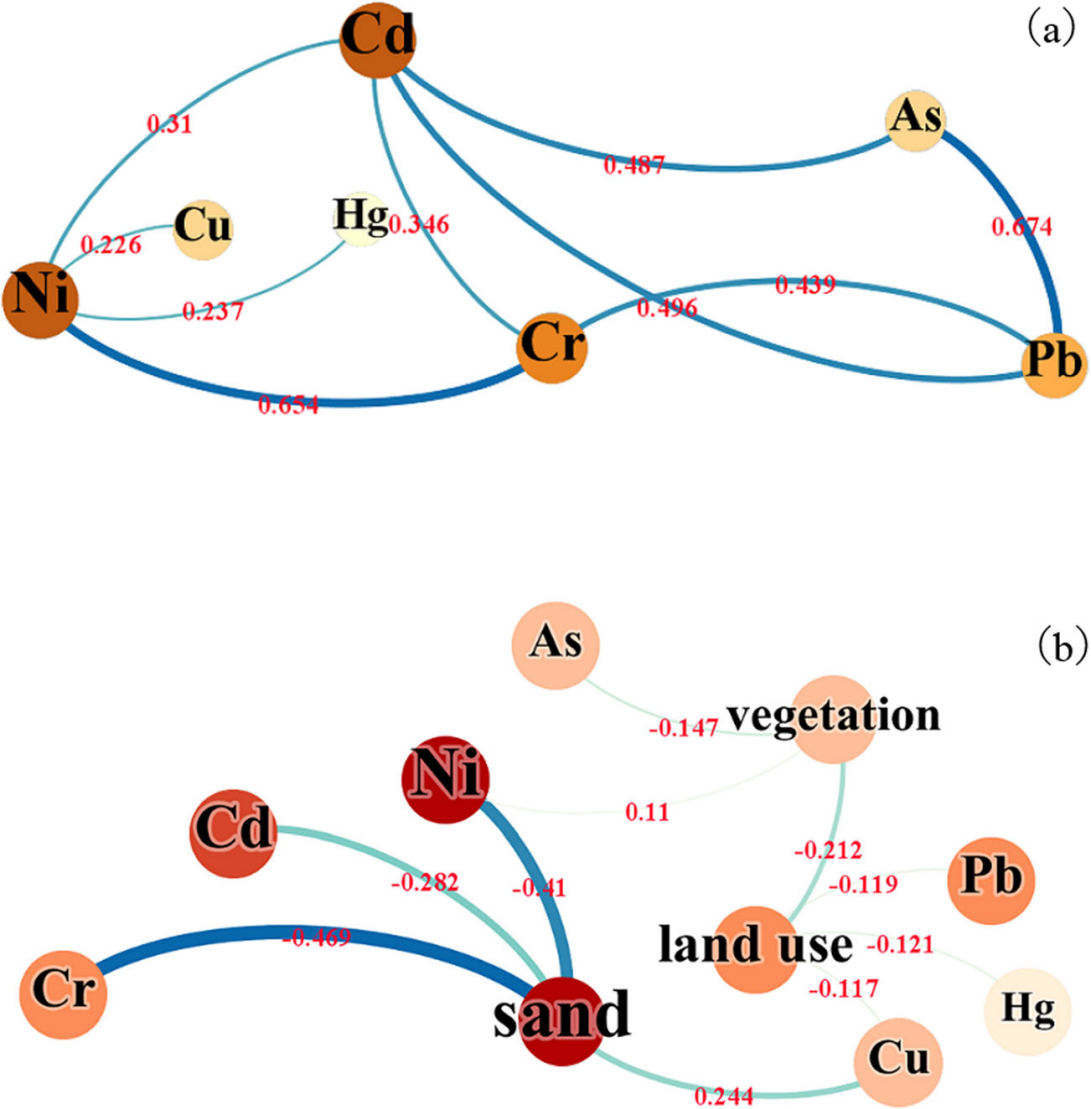
Co-occurrence network between soil heavy metals and soil physical and chemical properties, vegetation, and land use. The node label represents each index, the color depth and node size are proportional to the correlation (p-value is 0.01), The side shows the correlation between the indicators, and the thickness and color depth is proportional to the Pearson correlation coefficient

The co-occurrence network shows the Pearson correlation coefficient between heavy metals and soil texture. As seen from Fig. 4a, Cd, Pb, As are all positively correlated, and Ni, Cd, Cr are all positively correlated. Besides, Cr has a significant positive correlation with Pb, with a p-value of 0.439, and Ni has a positive correlation with Cu and Hg, and other elements are stronger than Ni. Among them, As element and Pb element and nickel element and chromium element have a significant correlation, p-values are 0.674, 0.654, copper element and mercury element are relatively less correlated with other heavy metal elements, and only have a weak positive correlation with nickel element One of the potential reasons for this situation may be the long-term stacking of local coal gangue [22–24]. It relates cadmium and nickel to many elements, which shows that their pollution sources are likely to be consistent.

It can be seen from Fig. 4b that it does not relate heavy metal elements to clay and powder, but are more related to grit. The reason for this phenomenon may be: the soil texture in the study area is mainly gritted, and the soil texture is heavier. Therefore, the leaching materials including heavy metals are poor, and the mass fraction kept in the soil is relatively high; at the same time, the adsorption performance of the soil colloid with heavier particle size is relatively relative, and the more heavy metal elements on it [25]. Grit is positively correlated with Cu and negatively correlated with Cd, Ni, Cr, and the correlation coefficients are 0.244, -0.282, -0.41, and − 0.469, respectively, showing that the texture of gravel is more likely to accumulate nickel and chromium. Temperate grasses, mosses, and miscellaneous grass swampy meadows As, Ni have a strong correlation with vegetation types, especially in the temperate grassy grassland. The three heavy metals Cu, Hg, and Pb have a strong negative correlation in the middle-top coverage grassland, showing that these heavy metals may have similar sources.

### Autocorrelation analysis

**Fig. 5:**
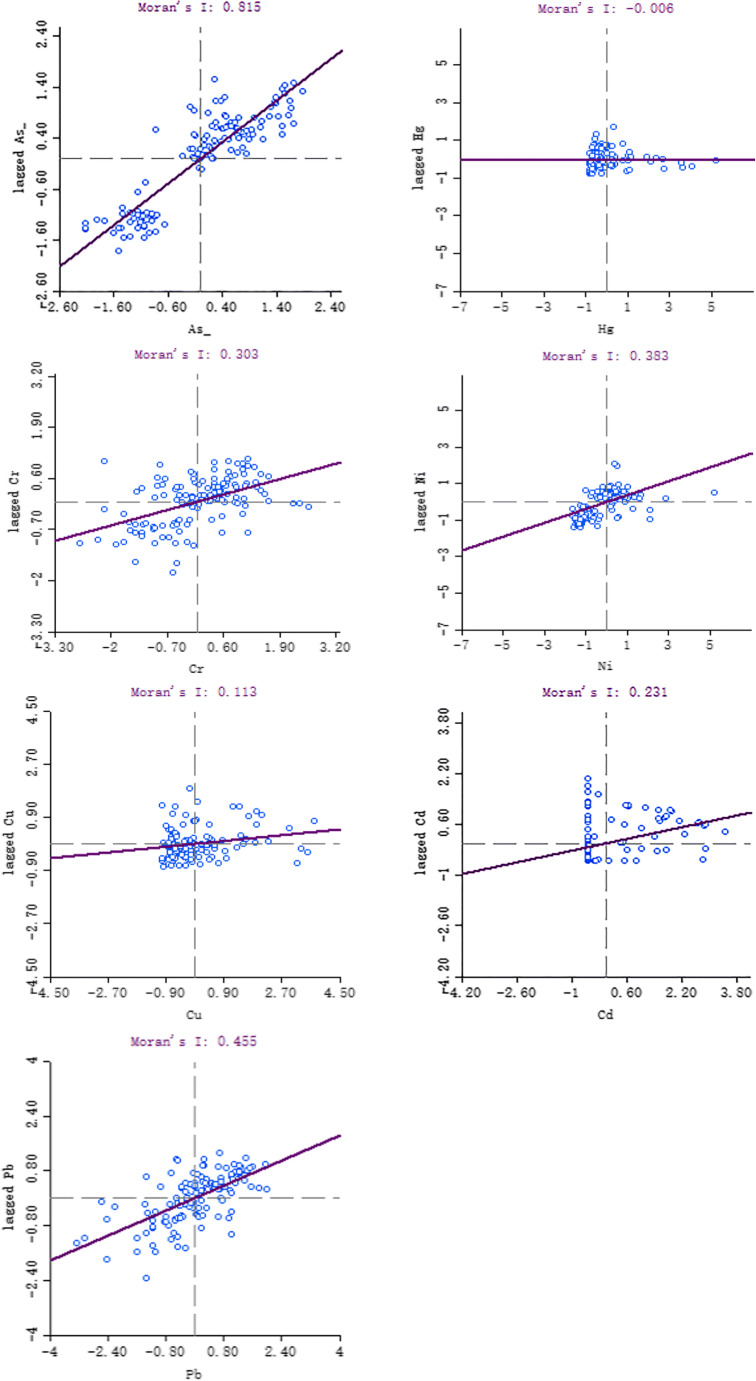
Moran’s I scatter plot

Regarding Fig. 5, the P-value is kept within the range of < 0.05, where arsenic, cadmium, chromium, nickel, and lead have significant spatial positive correlations, showing that the spatial distribution of heavy metal levels in the mining’s soil area does not show complete randomness. Rather, it shows the spatial agglomeration between similar values. As the spatial distribution position (distance) gathers, the correlation becomes more significant, and the spatial correlation of arsenic is the most obvious (Molan index is 0.815). Combined with various metal concentration levels, cadmium, nickel, and lead it to dis-distribute tributes concentrated areas with pollution sources. Although arsenic and chromium have obvious autocorrelation (the Moran index is 0.815, 0.303, respectively), their concentrations do not exceed the standard, and the concentration levels are mainly affected by natural the influence of background, not the influence of human activities. The distribution of mercury shows a negative correlation, and its value is close to 0, showing that because of the different development levels and structures in the three mining areas, the mercury element shows a large spatial difference. Most of the regional units are in the first and third quadrants and belong to the low-low aggregation and high-high aggregation types, showing that the high-observation area units are surrounded by the same high-value areas, and the low-observation area units are the same low-value Surrounded by the area, the heavy metal content of the adjacent area units is similar.

The spatial distribution characteristics of soil heavy metals in the study area were analyzed by the classical Bayesian Kriging difference method, and they converted the discrete data into spatial continuous data.

**Fig. 6:**
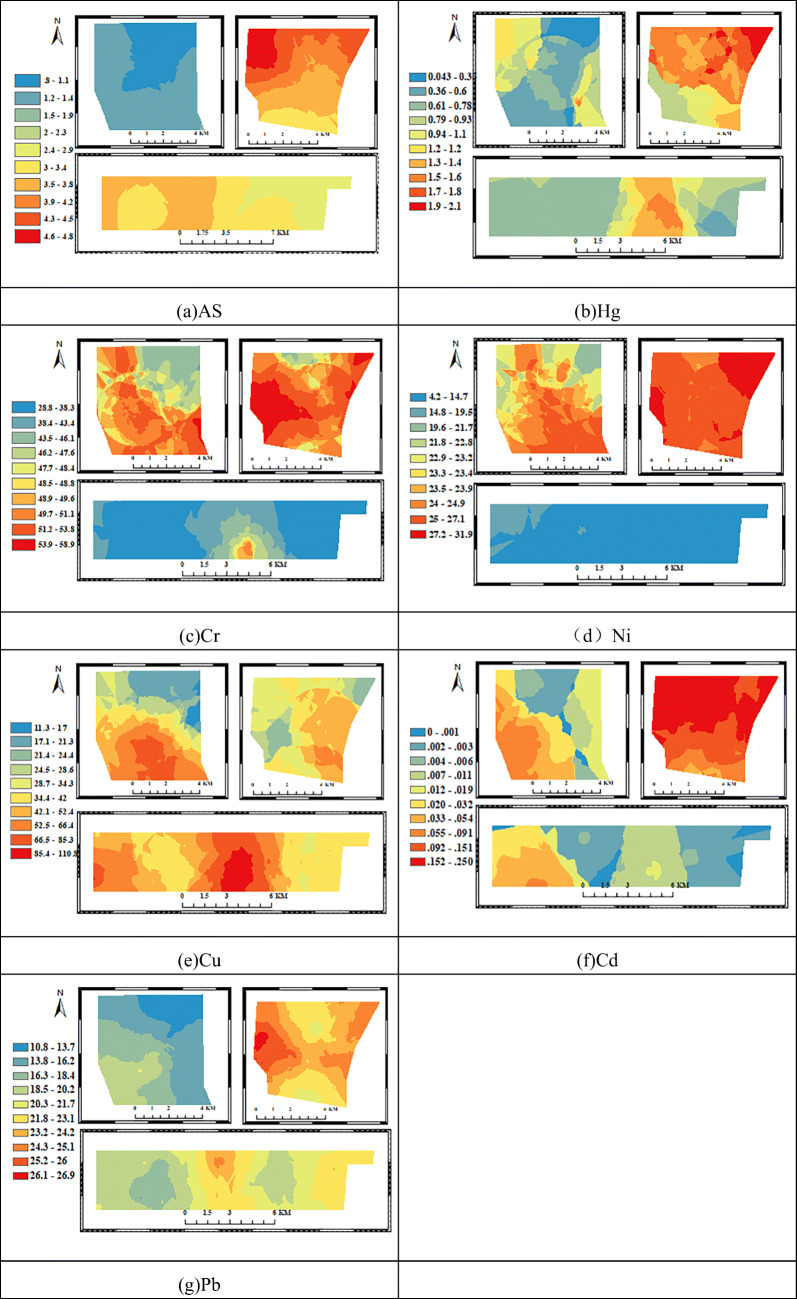
Spatial distribution of heavy metals in the study area

The high-arsenic areas are mainly concentrated in the PS and TS mining areas, and the spatial agglomeration phenomenon is obvious (Fig. 6). The most valuable area (4.6 mg/kg ~ 4.8 mg/kg) appears in the northwest of the TS mining area, which is lower than the local environmental background value, and the arsenic pollution is not obvious from the principal source of the arsenic element is influenced by the parent material [5, 24]. Among the heavy metal elements studied, the lead with the highest correlation with arsenic appeared to a comparable spatial distribution. The difference is that the chief lead value range can reach 26.1 mg/kg ~ 26.9 mg/kg, and is above the local background value (17 mg /kg), the TS mining area is affected by lead pollution. The lead element mainly comes from human mining activities. During mining, stacking, and transportation, a sizeable amount of dust is discharged, and it generates wastewater. It releases some pollutants into the nearby soil, resulting in lead pollution [12, 25, 26]. Relatively, the lead concentration is higher in the eastern part of the PS mining area, while the western region and the ES mining area are less affected by lead pollution [14, 27, 28]. Similarly, the concentration of cadmium in the TS mining area is the highest, and the entire mining area is above 1 mg/kg. The high value mainly occurs in the coal gangue stacking area and the farmland of the mining area. I concentrate on the cadmium in the ES and PS mining areas in the east, and the concentration in the west is relatively low. The difference in this content shows that it closely relates human mining activities are close to the distribution of heavy metals in the soil. The hotspots of mercury are still distributed in the TS mining area and are distributed diagonally in the northwest and southeast of the ES mine, and the east of the PS mine. The spatial distribution of chromium and nickel is similar, with the main difference being: the spatial diversity of the nickel element in the PS mine is small, accumulated in the range of 4.2 mg/kg ~ 16.9 mg/kg, fully within the allowable background value, and the chromium element is present in the eastern part of the region. The distribution of copper elements is uniformly distributed in the three’s south mining areas. The heavy metal level of PS is low, and the hot spots are mainly in the east-central part of the central region where the population is dense and there are many settlements. Human activities have a greater influence on the soil. From the above analysis, the high-value areas of heavy metals are distributed in the rest of the TS mining area except the southeast, and I concentrate on the population of the TS mining area in the southeast. This shows that human life has relatively little impact on the local heavy metals, and the principal factors are mining and coal preparation coal, and heavy metal accumulation in coal mine soil was the main cause [27, 29, 30].

## Conclusions

The concentration of most heavy metals is greatly affected by mining activities, leading to an increase in their average concentration. The results showed that 91 % of the mercury concentration data, 70 % of the chromium concentration data, 60 % of the nickel concentration data, 75 % of the copper concentration data, and 80 % of the lead concentration data in the three mining areas exceeded the local environmental background values (in order: 0.04 mg/kg, 41.4 mg/kg, 19.5 mg/kg, 14.1 mg/kg, 17.2 mg/kg). The co-occurrence network between heavy metals and soil texture shows that there is a significant positive correlation between As element and Pb element and between nickel element and chromium element. The p values are 0.674 and 0.654 respectively. The pollution sources are similar. Gravel and Cu are positively correlated. Cd, Ni, Cr are negatively correlated, and the correlation coefficients are 0.244, -0.282, -0.41, -0.469, respectively, indicating that the gravel texture is more likely to accumulate nickel and chromium. The Moran index is 0.815 (As), -0.006 (Hg), 0.303 (Cr), 0.383 (Ni), 0.113 (Cu), 0.231 (Cd), 0.455 (Pb). The combined spatial distribution shows that arsenic, cadmium, chromium, nickel, and lead have a significant positive spatial correlation, and the spatial agglomeration is obvious. The level of PS heavy metals is low, and the hot spots are mainly in the east-central area, where the population is dense and there are many settlements, and human activities have a greater impact on the soil. The TS mining area has a high level of heavy metals. The high-value areas are distributed in the northeast, southwest, and northwest, while the population is concentrated in the southeast, indicating that human life has relatively little impact on local heavy metals. The main factors are mining area mining and coal stacking, and coal mining soil The accumulation of heavy metals accounts for a major reason.
